# Descriptive Study of Children’s Nutritional Status and Identification of Community-Level Nursing Diagnoses in a School Community in Africa

**DOI:** 10.3390/ijerph17176108

**Published:** 2020-08-21

**Authors:** Pedro Melo, Maria Isabel Sousa, Matilde Mabui Dimande, Sónia Taboada, Maria Assunção Nogueira, Carlos Pinto, Maria Henriqueta Figueiredo, Tam H. Nguyen, José Ramón Martínez-Riera

**Affiliations:** 1 Universidade Católica Portuguesa, Institute of Health Sciences/School of Nursing (Porto)/Centre for Interdisciplinary Research in Health, 4169-005 Porto, Portugal; 2 Center for Health Technology and Services Research, NursID Project, 4200-450 Porto, Portugal; henriqueta@esenf.pt; 3 Universidade Católica Portuguesa, Institute of Health Sciences/School of Nursing (Porto), 4169-005 Porto, Portugal; isabel75ocua@gmail.com; 4 Maputo Central Hospital, Board of Directors, Maputo 1100, Mozambique; matimabui@yahoo.com.br; 5 SC Fitness, Sonae Capital, 4471–907 Maia, Portugal; sonia.taboada@scfitness.pt; 6 Instituto de Investigação e Formação Avançada em Ciências e Tecnologias da Saúde (IINFACTS), Cooperativa de Ensino Superior Politécnico e Universitário (CESPU), 4585-116 Gandra, Portugal; assuncaonog@gmail.com; 7 ACeS Porto Ocidental, North Region Health Administration, 4000-447 Porto, Portugal; carlospintorda@gmail.com; 8 Escola Superior de Enfermagem do Porto, 4200-072 Porto, Portugal; 9 William F. Connell School of Nursing, Boston College, Newton, MA 02467, USA; tam.nguyen@bc.edu; 10 Departamento Enfermeria Comunitaria, Medicina Preventiva y Salud Publica e Historia de la Ciencia, Universidad de Alicante, E-03080 Alicante, Spain; jr.martinez@ua.es

**Keywords:** nutritional surveillance, public health, community health nursing, public health nursing, children’s health, community participation

## Abstract

Effectively responding to children’s nutritional status and eating behaviors in Mozambique requires a community-based care approach grounded in sound nursing research that is evidence-based. The Community Assessment, Intervention, and Empowerment Model (MAIEC) is a nursing theoretical model that is based upon clinical decision-making for community health nurses using communities as a unit of care. We used the MAIEC to identify a community-based nursing diagnosis to address children’s nutritional status and eating behaviors in Mozambique. Objectives: (1) to conduct a descriptive study of children’s nutritional status and eating behaviors in a school community in Mavalane, Mozambique, and (2) to identify a community-based nursing diagnosis using the MAIEC clinical decision-making matrix in the same school community. Method: a cross-sectional, quantitative study was conducted to assess the nutritional status of children using anthropometric data, including brachial perimeter and the tricipital skinfold, and standard deviation for the relation of weight–height, in a sample of 227 children. To assess community management of the problem and identify a community-based nursing diagnosis, we surveyed 176 parents/guardians and 49 education professionals, using a questionnaire based on the MAIEC clinical decision matrix as a reference. Results: malnutrition was identified in more than half of the children (51.3%). We also identified a community-based nursing diagnosis of impaired community management related to the promotion of child health and healthy eating evidenced by the lack of community leadership, participation, and processing among more than 70% of the community members (parents/guardians and education professionals). Conclusion: a nursing diagnosis and diagnostic criteria for nutritional status and community management were identified. The need to intervene using a multidisciplinary public health approach is imperative, with the school community as the unit of care. In addition, reliable anthropometric data were identified as important criteria to complement the nursing diagnosis and guide future public health interventions.

## 1. Introduction

**Figure 1:**
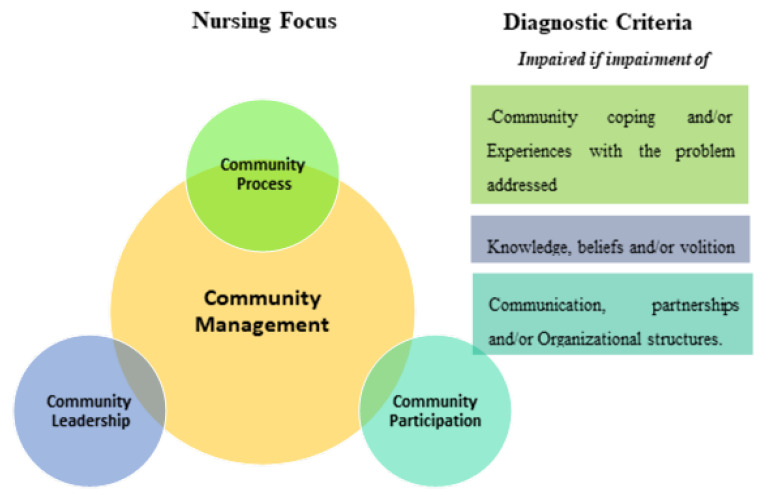
Clinical Decision Matrix components from Community Assessment, Intervention and Empowerment Model (MAIEC) [1,2,3].

Framing the community as a unit of care (i.e., approaching the whole community as a client) and promoting community empowerment as both process and outcomes are the forming principles of the Community Assessment, Intervention and Empowerment Model (MAIEC) [1,2,3]. This nursing model has a clinical decision matrix that guides nurses’ decision-making in relation to the community as the nursing client, Figure 1:

Worldwide, nurses have an international classification system, called the International Classification for Nursing Practice (ICNP) [4]. ICNP provides an agreed set of terms that can be used to record the observations and interventions of nurses across the world. ICNP also provides a framework for sharing data about nursing and for comparing nursing practice across settings. The ICNP diagnostic term that is of central focus in the MAIEC is “Community Management”. This focus has three dimensions of diagnosis, which are also included in the ICNP:

- *Community leadership*—related to the community’s knowledge, beliefs, behaviors and volition in the context of the problem addressed;
- *Community participation*—related to communication, partnerships and the existence of organizational structures;
- *Community process*—related to community coping or experiences with the problem addressed.

MAIEC also has the definitions of Community, Community Health, Community Environment and Community Health Nursing Care, framing, as metaparadigmatic concepts in Nursing, the assumptions (which are the premises that guide the use of the model) and postulates (which are the unquestionable principles that support the model) that support this theoretical nursing model [3].

Aligned with the strategic plan for the health sector in Mozambique (2014–2019), which supports the search for better solutions for health, with the involvement of communities, this research was integrated in the Nursing Research Platform of the Centre for Interdisciplinary Research in Health at the Universidade Católica Portuguesa. In this platform the MAIEC project is included, which aims to study the impact of this nursing model in community health gains and community empowerment. The MAIEC and its clinical decision matrix [1,2,3] guided this study to foster a community-based approach to local health problems.

The community that was the target of our research and care was a school community in the neighborhood of Mavalane. In this place there is an institution, created by the Missionaries of Good News, whose objective is to support families and children in difficult and vulnerable situations. Despite the contribution of some economic development, indicated by the Republic of Mozambique, Mozambique remains one of the poorest countries in the world, with 46% of Mozambicans living below the poverty line [5,6].

According to the strategic health plan [5], one in six children dies before reaching 5 years of age. The infant and youth mortality rate is 178 per thousand inhabitants and malnutrition is responsible for approximately 20% of deaths. Mortality levels are exacerbated mainly by poverty, low literacy of mothers and the precarious supply of drinking water and basic sanitation [7]. Nutritional indicators report that 43% of children under 5 years old suffer from moderate chronic malnutrition and 20% suffer from severe chronic malnutrition [7]. A recent study highlighted the importance of articulating policies and interventions at the local level, as well as training health and education professionals in promoting healthy eating in children [8]. However, local information about malnutrition and community resources in Mavalane are not sufficiently available to effectively guide interventions. Moreover, there is a lack of research using a community-based nursing model, such as the MAIEC, to provide a strong theoretical basis for future interventions. Last, the problem of malnutrition and community management has not been well articulated within the context of ICNP, which can help provide consistent language and a framework for interventions in this setting and beyond.

Regarding these public health problems related to child nutrition and unhealthy eating (the use of foods with low nutritional value or with inappropriate cooking) in Mozambique and the evidence of a nursing model that allows an objective diagnosis in community [1,2,3], we believe that community-based research should be developed. This research will promote the implementation of nursing science (using nursing process as a resource) to the two phenomena: the nutritional status and the community management in order to answer this problem.

Regarding nutritional status, studies related to nursing decision making were not found. As we are using ICNP as a nursing classification reference, it is appropriate to introduce the definition of nutritional status into this classification. According to ICNP, nutritional status represents “Weight and body mass in relation to intake of nutrition and specific nutrients estimated according to height, body build and age.” [4]. Specifically related to the nutritional status of children, most of the studies found suggest as good indicators the anthropometric data, such as brachial perimeter, tricipital skinfold, and the calculation of brachial mass area and brachial fat area. The researchers also suggest as good indicators the Z-scores and the Composite Z-scores [9,10,11,12,13,14]. This information should be complemented with the evaluation of the Standard Deviation in the Weight–Height ratio in children under the age of 5 years and the standard deviation in the Body Mass Index (BMI) for children over the age of five, according to reference values, in the case of this study, published by the Mozambique Health Authorities [15,16].

Regarding Community Management, MAIEC’s clinical decision matrix allows the adequation of the data to the problem of children’s nutritional status and healthy eating [1,2,3], and the questions must be defined in a process of expert consensus.

Therefore, the aims of this study are:

- To describe the “Nutritional Status” of school children in the community of Mavalane, Mozambique using anthropometric data;
- Identify a nursing diagnosis focused on “Community Management” related to children’s nutritional status and healthy eating, using the MAIEC clinical decision-making matrix [1,2,3] in the same school community in Mavalane, Mozambique.

## 2. Materials and Methods

The study was developed in a community of schools that provide education to children from 1 to 6 years old, in the Mavalane neighborhood in Mozambique, Africa.

To assess the nutritional status of children, a cross-sectional, quantitative study was conducted using anthropometric data, including the brachial perimeter (BP) and the tricipital skinfold (TS). From this data, brachial mass area (BMA), brachial fat area (BFA), and the respective Z-scores and Composite Z-scores were calculated, as proposed by different researchers [9,10,11,12,13,14], as a sensitive method to assess nutritional status in relation to the anthropometric data considered. The collection of these data, not being invasive and having a low cost, were considered ideal for the context of the study since our resources were scarce, namely technological resources like Bioelectrical Impedance Analysis machines. The standard deviation in the Weight–Height ratio in children under the age of 5 years and the standard deviation in the Body Mass Index (BMI) for children over the age of 5 was added to the Z-scores, according to reference values for Mozambique [15,16].

The collection of data was conducted by one of the researchers, a student from the Master’s in Nursing course at Universidade Católica Portuguesa (that is a nurse with a bachelor’s degree completing a master’s degree in nursing, with specialization in Community and Public Health Nursing). The student was orientated to a children and family approach in response to communication from the Nurse-Director from Maputo Hospital. Additional training from a researcher, in Portugal, was provided in the area of nutrition, to ensure that the student had all the skills needed to assess the anthropometric data. Universidade Católica Portuguesa provided all the necessary material for the assessment (adipometers and forms).

To the 566 children who attended the schools in the project’s partner institution in Mavalane, the following inclusion criteria were applied:

- They attend schools included in the study.
- They have explicit authorization from parents or guardians to be included in the study, in the informed consent form.
- They accept to be evaluated.

The recruitment of children, families, and education professionals was made through the schools’ directors, who introduced potential participants to the nurse researcher. The nurse researcher made a first meeting with the parents/children’s guardians and school professionals locally, to describe the study, invite them to participate, and provide informed consent forms. Children, parents/guardians, and educational professionals were included in the study if they met all the inclusion criteria and signed the informed consent.

With these criteria, anthropometric data was collected from 227 children. Each child was assessed in a private room close to the classrooms by the nurse researcher to ensure privacy as well as emotional and physical security.

For aim 1: The following diagnostic criteria, based on expert consensus between the researchers’ team-nurses and nutritionist, were used to describe the nutritional status of children in our study:

- The Z-scores and composite Z-scores, proposed by the studies used by reference [9,10,11,12,13,14] D.
- The standard deviation in the Weight–Height ratio (for children bellow 5 years old) or standard deviation in the Body Mass Index (for children over 5 years old), proposed by Health Authorities in Mozambique, as described in Table 1:

For the evaluation of the community management focus for the promotion of healthy eating in children, a questionnaire was created, by a process of expert consensus with the project researchers, based on the MAIEC clinical decision matrix, and administered to the leaders and members of the affected community.

The inclusion criteria for community leaders were:

- Being a coordinator/director of the assessed schools.
- Accepting to participate in the study explicitly with a signed informed consent form.

The inclusion criteria for community members were:

- Being a parent or guardian or education professional of the children that attend the assessed schools.
- Accepting to participate in the study explicitly with a signed informed consent form.

The questionnaire included two parts. The first part focused on participants’ sociodemographic characterization, which included the realization of a genogram and the adapted Graffar scale proposed by Figueiredo [17] applied to parents. The second part of the survey had questions related to the diagnostic dimensions of community management, including:

(a) Community Leadership (knowledge, beliefs and behaviors associated with children’s health and nutrition—Associated with the parental role in parents and the professional role in the education and health professionals involved).
(b) Community Participation (perception of the existence of organizational structures and partnerships to promote healthy eating for children).
(c) The Community Process (previous experiences with health and food promotion projects).

A pre-test of the questionnaire was carried out on members of a school community with characteristics similar to the population under study (9 education professionals and 20 parents), to assess for clarity and relevance. This school community was excluded from the posterior study. Based on the pre-test, no revisions to the original version were proposed. When we reassess the community management in one to two years, after we develop and implement the intervention, we will be able to assess the questionnaire’s reliability and validity and publish the results at that time. However, we considered the data that resulted from the questionnaire to make a nursing diagnosis that allowed an epidemiological image of the state of community management based on MAIEC.

The questionnaire was administered to 176 out of 227 (77%) parents of the evaluated children and 49 out of 52 (94%) education professionals of the evaluated schools. The data were analyzed with Microsoft Excel 2007. The statistical analyses considered the measure of proportion of responses in relation to the diagnostic dimensions proposed by the clinical decision matrix of the MAIEC, as described above.

The study was submitted to the ethics committee of the Institute of Health Sciences of the Portuguese Catholic University, which gave a favorable opinion—CE.C. (10) 2018. Informed consent was obtained from all parents and children, with communication adapted to the children’s age. The study ran from September 2018 to January 2019.

## 3. Results

We now present the results related to nutritional status and community management, starting with the first one:

### 3.1. Nutritional Status According to Age and Sex

The ages of the 227 children evaluated had a minimum of approximately 2 years (23 months) and a maximum 5 years and 9 months. The average age was approximately 4 years (51.24 months). Forty seven percent (107) of the children were male and 53% (120) were female.

Table 2 shows the results of the Z-scores for the different parameters assessed individually, as well as the evaluation of the Z-scores for the set of parameters (composite Z-scores), related to nutritional status.

Considering the statistical analysis related to the percentage distribution of the Z-scores and also the composite Z-scores identified for the different anthropometric measures (which allows an integrated analysis of the nutritional status from all the analyzed parameters), we found that 51.3% (*n* = 119) of children had a moderate malnutritional status (composite Z-score between −2 and 0). Our study identified 10.7% of children with moderate malnutrition.

In relation to children above average the weight parameters, we identified only one child (0.4%) with a composite Z-score above three and two children (0.8%) with a composite Z-score between two and three.

To these data we added the analysis of the percentage distribution related to the weight/height ratio for children under 5 years old and the Body Mass Index for children older than 5 years.

One hundred and sixty-five children under 5 years old were evaluated, which corresponds to 72.7% of the total children evaluated. Table 3 shows the data relating to the Standard Deviation for the Weight–Height ratio, according to the reference values for Mozambique:

In children under 5 years of age, it appears that 7.8% had values of Standard Deviation in the Weight–Height ratio (SD W–H) below “−2” and therefore had a state of malnutrition present in a very high degree. However, 10.9% had values for SD W–H above two, indicating over nutrition.

In children over 5 years old, we analyzed the standard deviation for the BMI (SD BMI/age), according to the reference values for Mozambique. Sixty-two children older than 5 years were evaluated, representing 27.3% of the total children evaluated.

Regarding the BMI/age SD, related to children older than 5 years, the values found are shown in Table 4:

From the analysis of the data presented in Table 4, it was identified that 9.6% of children over 5 years of age presented a state of malnutrition present in a very high degree (SD BMI ≤ −2) and 1.6% had over-nutrition (SD BMI = 2).

According to all the data, the following descriptive assessment can be made about children’s nutritional status:

- *Severe malnutrition status* was detected in 7.8% of children under five years old and 9.6% of children older than five, but considering the whole sample and the Z-scores of the anthropometric data assessed this diagnosis had an expression of 1.3% and 0, when we considered the composite Z-scores.
- *Moderate malnutrition status* was detected in 13.3% of children under five years and 0% in children over 5 years old. Considering the whole sample and the composite Z-scores, 10.7% of the children were identified with this nursing diagnosis.
- *Low malnutrition status* was detected in 3.7% of children under five years old and 16.1% of children older than five years old. Considering the composite Z-scores this diagnose represented 40.6% of the whole sample of children.
- *Normal nutritional status* was detected in 55.8% of children under five years and 54.8% of children above five years old. However, considering the composite Z-scores of anthropometric data this percentage fell to 40% of the children assessed.
- *High overweight* was detected in 12.1% of children under five years old and 17.7% of children over five years old. Considering composite Z-Scores this percentage fell to 7.5% of the sample of children.
- *Very high overweight* was detected in 10.9% of children under five years old and in 1.6% of children above five years old. According to the composite Z-scores of anthropometric data this diagnose represented 0.8% of children.
- *Extreme high overweight* was detected in none of children, considering the standard deviation for Weight–Height Ratio in children under five and BMI/age for children above five. However, considering the composite Z-scores of anthropometric data, a percentage of 0.4% of children with this nursing diagnosis was found.

### 3.2. Assessment of Community Management

In the context of the community management focus, 49 education professionals were surveyed, of which 98% (48) were female and the majority (44.9%, *n* = 22) were in the 30–39 age group.

Regarding educational qualifications, 44.9% (*n* = 22) had between the 11 and 12 years of education and 64.2% (*n* = 27) had from the eight to the twelve years of schooling. Fifty-nine percent of the education professionals had the category of child educator, which requires specific training in the area of early childhood education.

Relating to parents, from the 176 assessed, 45% (*n* = 79) were between 20 and 29 years old, followed by 35% (*n* = 62) between 30 and 39 years old, and 20% (*n* = 35) below 20 years old. From the analysis of genograms and Graffar scales, it was found that 61% of the sample of parents refers to extended families with a predominance of lower middle class (52%, n = 91) and low class (19%, n = 33).

Regarding the nursing diagnosis, we identified the diagnosis of impaired community management. Table 5 presents the whole nursing diagnosis focused on community management, concerning the three diagnostic dimensions proposed by MAIEC, and all the sub-diagnoses and identified in the community (parents/children guardians and educational professionals):

## 4. Discussion

The assessed data allowed the identification of, according to the diagnostic criteria defined by the research team, different nursing diagnoses, using ICNP as a reference [4]. It is also evident that the parameters used by health services to assess children health concerning to nutritional status (weight-height and BMI standard deviation), can be complemented with the anthropometric parameters used in this study, because they gives a deeper analysis of nutritional status, beyond the weight/height relation. The studies that were references for this research are, in this way, confirmed as good orientations to be used as complementary methods to assess nutritional status in children in a public health perspective and to identify nursing diagnoses with this ICNP focus. In relation to this statement, we emphasize the guidelines of Mascarenhas and his collaborators [9] or Frisancho [10,11], who emphasize the importance of using the anthropometric data identified in our study, as important indicators of children’s nutritional status, when researchers have few resources. In addition, Monteiro [12], Hoffman [13] or Bechard [14] and their collaborators, regarding children with more vulnerable health conditions, present the anthropometric data used in our study, which do not require invasive assessment techniques, as important indicators of children’s nutritional status. The interdisciplinary approach between nurses and nutritionists was identified as important in the research approach that promoted the dialogue between these two professionals in the discussion of diagnostic criteria to assess children’s nutritional status.

As a priority in public health in Mozambique with a relevant impact on child morbidity and mortality, the data support the important need to intervene in improving the nutritional status of children, that has been diagnosed as problematic. The problem of overnutrition and severe malnutrition identified in children below 5 years old, suggests a need to intervene at lower ages in the prevention of both problems, considering as hypothesis that this can have impact in older children’s nutritional status moving forward. Considering the data from the Mozambique authorities, relating children mortality with community-based problems [7], the assessment of a community-based diagnosis, using MAIEC was important [1,2,3].

The assessment of community management (together with members and leaders of school communities) to promote healthy eating and skills to assess the problem, revealed a serious impairment in several dimensions. Both in parents and in education professionals, there were very high knowledge deficits about adequate nutrition and children’s health surveillance (over 70% in all dimensions evaluated and in some cases over 90% of members and leaders assessed). The behavioral dimension was also identified as being impaired to a very high degree, both in the children’s parents and in education professionals, with inadequate eating behaviors and equally inadequate monitoring of eating habits.

The lack of organizational structures and partnerships in the community to promote adequate eating in children and children’s health intensifies the problem. Taken together, the following nursing diagnoses were identified: *impaired community management* for child health promotion, specifically concerning nutritional status, related to *impaired community leadership*, *impaired community participation*, and *impaired community process*. This diagnosis in the community allows the understanding that MAIEC was a useful model in approaching the community, allowing in an objective way the evaluation of community management, in a community-based approach to answer the problem under study.

This study has several limitations. Firstly, it is a cross-sectional and descriptive study. Therefore, the results relate to the sample studied and the period studied and are reflective of the children who participated in the study and attend the schools covered in the project. Multivariate analysis was not used to predict the impact of nutritional status on other variables under study, like the children’s age, family’s social status, etc. We intend to present these analyses in a posterior paper. Regarding the questionnaire for the evaluation of the community management focus, even though a pre-test was conducted, the cultural aspects do not guarantee the understanding of all questions in the same way by all participants in the study. Future studies will address this as well as assessing the reliability and validity of the questionnaire.

Despite these limitations, the study contributes to the profession of nursing, to the science of nursing and to society.

As contributions to the profession, this study provides a path and foundation for future interventions, based on a clinical decision that considers community as a unit of nurses’ care. In addition, collaborating with local political and organizational structures, including members and leaders from schools and surrounding organizations from the community, increases the chance of success for future interventions.

Interdisciplinary synergies were also promoted, based on public health and community health from the perspective of nursing and nutrition, in order to obtain health gains, namely those sensitive to community and public health nursing and nutrition care.

As contributions to society, this community-based research study improved citizenry and community participation, thereby strengthening the community’s resources to improve nutritional status and children’s health and addressing a local public health priority.

As contributions to the research, the application of a theoretical model of Nursing, MAIEC, was used on a problem where it had never been applied. This facilitated clinical decision making for nurses, in the context of the community as a client, related to the nutritional status and children’s health. Other experiences in other contexts have shown the advantage of considering the community as target of nursing care [18,19], but this project was innovative in relation to nutritional status and children’s health. In addition, using the MAIEC decision matrix to guide future community-based longitudinal intervention will increase the likelihood of improving community management, a central focus of the attention of community health nursing specialists.

Future research in this area must ensure that a multidisciplinary approach, between nursing and nutrition sciences, is used to monitor the nutritional status of children. To achieve this, the assessment of nutritional status should be repeated, using the same procedures and in the same sample, after applying the intervention proposed by MAIEC, leaving an interval of 1 to 2 years after its application to adequately assess the impact of the changes promoted in the community related to the nutritional status of children.

## 5. Conclusions

The diagnosis of the health status of a population, in this case of children from a school community in Mozambique, using a multidisciplinary approach, allowed the identification of an impaired nutritional status regarding its prevalence in the context of malnutrition and overnutrition. The multidisciplinary data analysis (nursing and nutrition) allowed the identification of the nursing diagnosis: impaired nutritional status, supporting it with the diagnosis of the nutrition area, from a public health perspective.

At the same time, the approach of the School Community as a care unit, in a systemic perspective and shaped by a theoretical nursing model, MAIEC, allowed the identification of the community health nursing diagnosis: impaired community management of the promotion of children health and healthy eating in children.

## Figures and Tables

**Table 1:** Nursing Diagnoses and Diagnostic Criteria for “Nutritional Status” in Children.

Nursing Diagnose	Diagnostic Criteria
Severe malnutrition status	Composite Z-score equal or below −2 and/or Standard Deviation in the Weight–Height ratio (for children bellow 5 years old) or Standard Deviation in the Body Mass Index (for children over 5 years old) below −2
moderate malnutrition status	Composite Z-score between −2 and −1 and/or Standard Deviation in the Weight–Height ratio (for children bellow 5 years old) or Standard Deviation in the Body Mass Index (for children over 5 years old) between −2 and −1
Low malnutrition status	Composite Z-score between −1 and 0 and/or Standard Deviation in the Weight–Height ratio (for children bellow 5 years old) or Standard Deviation in the Body Mass Index (for children over 5 years old) between −1 and 0
Normal nutritional status	Composite Z-score between 0 and 1 and/or Standard Deviation in the Weight–Height ratio (for children bellow 5 years old) or Standard Deviation in the Body Mass Index (for children over 5 years old) between 0 and 1
High overweight	Composite Z-score between 1 and 2
Very high overweight	Composite Z-score between 2 and 3 and/or Standard Deviation in the Weight–Height ratio (for children bellow 5 years old) or Standard Deviation in the Body Mass Index (for children over 5 years old) between 2 and 3
Extreme high overweight	Composite Z-score over 3 and/or Standard Deviation in the Weight–Height ratio (for children bellow 5 years old) or Standard Deviation in the Body Mass Index (for children over 5 years old) over 3

**Table 2:** Distribution of Z-Scores from Brachial Mass Area (BMA), Brachial Fat Area (BFA), Brachial Perimeter (BP) and Tricipital Skinfold (TS) and Composite Z-Scores.

Z-Score Ranges	ZBMA		ZBFA		ZBP		ZTS		Composite Z-Scores	
	fi	%	fi	%	Fi	%	fi	%	fi	%
−3.00 a −2.00	1	0.4	1	0.4	2	0.8	3	1.3	0	0
−2.00 a −1.00	35	15.2	27	11.7	35	15.2	27	11.7	23	10.7
−0.99 a −0.01	84	37.9	100	45.1	72	31.7	95	42.4	92	40.6
=0.00	0	0	0	0	0	0	0	0	3	1.3
0.00 a 1.00	68	29.7	71	31.2	83	36.4	77	33.9	88	38.7
1.00 a 2.00	31	13.5	22	9.2	29	12.7	17	7.3	18	7.5
2.00 a 3.00	8	3.3	4	1.6	5	2.1	5	2.1	2	0.8
>3.00	0	0	2	0.8	1	0.4	3	1.3	1	0.4
Total	227	100.0	27	100.0	272	100.0	27	100.0	227	100.0

Acronyms description: ZBMA-Z-score for Brachial mass area; ZBFA-Z-scores for Brachial Fat Area; ZBP: Z-scores for Brachial Perimeter; ZTS-Z-scores for Tricipital Skinfold.

**Table 3:** Percentage Distribution of Children Under 5 Years of Age, in Relation to the Standard Deviation in the Weight–Height Ratio.

Standard Deviation Weight-Height Ratio	Frequency	Percentage
−3.00 to −2.00	13	7.8
−1.99 to −1.01	6	3.7
−1.00 to −0.01	16	9.7
0	84	50.9
0.01 to 0.99	8	4.9
1.00 to 1.99	20	12.1
2.00 to 3.00	18	10.9
Total	165	100

**Table 4:** Percentage Distribution of Children Older than 5 Years Evaluated, in Relation to the SD BMI/Age.

SD BMI/Age	Frequency	Percentage
−3.00 a −2.00	6	9.6
−1.00 a −0.01	10	16.1
0.00	34	54.8
1.00	11	17.7
2.00	1	1.6
Total	62	100

SD BMI/age- Standart Deviation for Body Mass Index/age.

**Table 5:** Diagnostic Dimensions Diagnoses and Sub-Diagnoses Identified in the Context of the Administrative Management Focus.

Diagnostic Dimensions Diagnoses	Sub-Diagnoses
**Impaired Community Leadership**	Parental Role:
	Cognitive dimension
	Knowledge about healthy eating (food quality) not shown in 98% Knowledge of community resources to seek informational support about food not demonstrated in 85% Knowledge about caring for children with gastrointestinal disorders not demonstrated in 77% Knowledge about nutritional status assessment of children not demonstrated in 77%
	Behavioral dimension:
	Adherence behavior to adequate water intake by children not demonstrated: inadequate amount of daily water in 82% Adherence behavior to the appropriate number of meals not demonstrated in 100% Adherence behavior to food of adequate quality not demonstrated in 85% Adherence behavior to daily soup consumption by children not shown in 94% Adherence behavior to control the consumption of sweets by children is not adequate in 79%
	Professional Role (Education Professionals): Cognitive dimension:
	Knowledge about assessing children’s nutritional status not demonstrated in 92% of educator Knowledge about caring for children with diarrhea not shown in 71% of educators Knowledge about resources for information on food security not shown in 78% of educators Knowledge of suitable fruit portions for children not demonstrated in 94% of educators Knowledge about School Health Program content not shown in 57% of educators.
	Behavioral dimension:
	Adherence behavior to candy consumption control not demonstrated in 70% of educators
**Impaired Community Participation**	Inexistence of organizational structures to promote health and healthy eating in children, in the perception of 100% of the evaluated community members; Inexistence of Partnerships related to the promotion of health and healthy eating in children, in the perception of 100% of the evaluated community members Inexistence of a communication mechanism on the issue of child health and nutrition, in the perception of 100% of the evaluated community members
**Impaired Community Process**	Impaired community coping—no previous experience associated with children’s health problems and food perceived by 100% of community members.
